# RETRACTION: Capn4 is a Marker of Poor Clinical Outcomes and Promotes Nasopharyngeal Carcinoma Metastasis via Nuclear Factor‐κB‐Induced Matrix Metalloproteinase 2 Expression

**DOI:** 10.1111/cas.16305

**Published:** 2024-08-05

## Abstract

**RETRACTION**: P.‐C. Zheng, X. Chen, H.‐W. Zhu, W. Zheng, L.‐H. Mao, C. Lin, J.‐N. Liu and M. Zheng, “Capn4 is a Marker of Poor Clinical Outcomes and Promotes Nasopharyngeal Carcinoma Metastasis via Nuclear Factor‐κB‐Induced Matrix Metalloproteinase 2 Expression,” *Cancer Science* 105, no. 6 (2014): 630–638, https://doi.org/10.1111/cas.12416.

The above article, published online on 07 April 2014 in Wiley Online Library (wileyonlinelibrary.com), has been retracted by agreement between the journal Editor‐in‐Chief, Masanori Hatakeyama; Japanese Cancer Association; and John Wiley & Sons Australia, Ltd. Following publication, concerns were raised by a third party regarding suspected overlap of elements within the western blot presented in Figure 3b and a figure published earlier in another paper elsewhere. Furthermore, image analysis uncovered several instances of overlap within the cell migration and cell invasion Transwell assay images presented in Figure 3c. The authors were unable to provide the original data and did not provide a satisfactory explanation for the overlaps. As a result, the article's conclusions can no longer be considered valid.

**RETRACTION**: P.‐C. Zheng, X. Chen, H.‐W. Zhu, W. Zheng, L.‐H. Mao, C. Lin, J.‐N. Liu and M. Zheng, “Capn4 is a Marker of Poor Clinical Outcomes and Promotes Nasopharyngeal Carcinoma Metastasis via Nuclear Factor‐κB‐Induced Matrix Metalloproteinase 2 Expression,” *Cancer Science* 105, no. 6 (2014): 630–638, https://doi.org/10.1111/cas.12416.

The above article, published online on 07 April 2014 in Wiley Online Library (wileyonlinelibrary.com), has been retracted by agreement between the journal Editor‐in‐Chief, Masanori Hatakeyama; Japanese Cancer Association; and John Wiley & Sons Australia, Ltd. Following publication, concerns were raised by a third party regarding suspected overlap of elements within the western blot presented in Figure 3b and a figure published earlier in another paper elsewhere. Furthermore, image analysis uncovered several instances of overlap within the cell migration and cell invasion Transwell assay images presented in Figure 3c. The authors were unable to provide the original data and did not provide a satisfactory explanation for the overlaps. As a result, the article's conclusions can no longer be considered valid.

